# Early discontinuation of steroid treatment in children with abdominal pain due to IgA vasculitis

**DOI:** 10.1007/s00431-025-06107-7

**Published:** 2025-04-04

**Authors:** Sumika Kambara, Nobuhiro Nishio, Yuichiro Sugiyama, Yosuke Nishio, Yukina Takamoto, Fumie Kitai, Yuma Takahashi, Nozomi Hayashi, Kazunori Haruta, Maki Kondo, Naoko Oike, Takeshi Miwa, Nobuhiro Watanabe, Marei Omori, Fumie Kinoshita, Taiki Furukawa, Jun-ichi Kawada, Hiroyuki Kidokoro, Yoshiaki Sato, Yoshiyuki Takahashi, Nagoya Collaborative Clinical Research Team, Kazuto Ueda, Makoto Oshiro, Atsushi Tashiro, Kazuki Yamamori, Motohiro Shibata, Shinji Hasegawa, Naoko Nishimura, Masashi Morishita, Michio Suzuki, Tetsuo Kubota, Noriko Nagai, Osamu Shinohara, Satoru Doi, Mitsuharu Kajita, Shinya Hara, Takashi Kawabe, Shin Hoshino

**Affiliations:** 1 Department of Pediatrics, Nagoya University Graduate School of Medicine, Nagoya, Japan; 2 Center for Advanced Medicine and Clinical Research, Department of Advanced Medicine, Nagoya University Hospital, 65 Tsurumai-Cho, Showa Ward, Nagoya City, Aichi Prefecture 466-8560 Japan; 3 Department of Pediatrics, Japanese Red Cross Aichi Medical Center Nagoya Daiichi Hospital, Nagoya, Japan; 4 Department of Pediatrics, Meitetsu Hospital, Nagoya, Japan; 5 Department of Pediatrics, Hekinan Municipal Hospital, Hekinan, Aichi Japan; 6 Data Coordinating Center, Department of Advanced Medicine, Nagoya University Hospital, Nagoya, Japan; 7 Medical IT Center, Nagoya University Hospital, Nagoya, Japan; 8 Division of Neonatology, Center for Maternal-Neonatal Care, Nagoya University Hospital, Nagoya, Japan

**Keywords:** IgA vasculitis, Prednisone, Steroid dosage, Treatment duration

## Abstract

This study aims to evaluate the impact of early steroid discontinuation on total dosage and outcomes in pediatric immunoglobulin A (IgA) vasculitis patients with uncontrolled abdominal pain. This retrospective cohort study included children younger than 16 years with newly diagnosed IgA vasculitis hospitalized for abdominal pain who received their first dose of steroids between April 1, 2013, and March 31, 2019, at 14 hospitals. Patients were divided into two groups: the standard (STD) group, which received steroid therapy for at least 8 consecutive days, and the early discontinuation attempt (EDA) group, which attempted discontinuation within 7 days. EDA was further divided into two subgroups: the early discontinuation (ED) group, which completed steroid treatment within a week, and the readministration (RA) group, which required readministration. Total steroid dosage, duration of therapy, hospital stay, and complications were compared. A total of 272 patients were analyzed: STD (*n* = 190) and EDA (*n* = 82). There were no significant differences in baseline characteristics. EDA had a shorter hospital stay (8.5 vs. 15.0 days, *p* < 0.01), fewer total steroid days (6 vs. 17.5 days, *p* < 0.01), and lower total steroid dosage (5.4 mg/kg vs. 15.4 mg/kg, *p* < 0.01) compared to STD, with no significant differences in complications. Among EDA patients, 22 (27%) required steroid readministration due to symptom recurrence; however, symptoms resolved in all RA patients, with lower total steroid dosage and duration compared to STD, without prolonging hospital stay.

*Conclusion*: Discontinuing steroids within 7 days for abdominal pain in children with IgA vasculitis reduces total steroid dosage without increasing complications, even with occasional readministration.

*Clinical trial registration*: Approval no. 2019–0394.

**What is known:**

• *Steroids have been reported to be effective for abdominal pain in pediatric IgA vasculitis.*.

• *Steroids should be tapered gradually to reduce the risk of symptom flare-up in pediatric IgA vasculitis.*.

**What is new:**

• *Early discontinuation of steroids reduced total dosage and hospital stay without increasing complications in pediatric IgA vasculitis.*.

## Introduction

Immunoglobulin A (IgA) vasculitis, formerly known as Henoch-Schönlein purpura, is a systemic small vessel vasculitis characterized by palpable purpura, arthritis, abdominal symptoms, and nephritis [1]. The complication rate of gastrointestinal lesions in IgA vasculitis is reported to be 50–80%, with abdominal pain being the most common symptom [2, 3]. For many years, steroids have been reported to be effective for severe abdominal pain in pediatric IgA vasculitis [4–8]. However, there is a wide range of reports on the dosage and duration of steroid administration, and there is still no evidence-based but only consensus- or opinion-based recommendation on the dosage and duration of administration.

Early steroid discontinuation may be beneficial for patient burden and medical costs by shortening the hospital stay. However, due to the fear of symptom relapse, no reports are currently evaluating the effectiveness of early steroid discontinuation. The purpose of this study is to assess the effect of early steroid discontinuation in patients with pediatric IgA vasculitis-associated abdominal pain.

## Methods

### Study design, setting, and data sources

This study is a multi-center retrospective cohort study. Eligible patients were children younger than 16 years who were hospitalized for newly diagnosed IgA vasculitis and received the first dose of steroids for uncontrolled abdominal pain between April 1, 2013, and March 31, 2019, at 14 general hospitals in the Aichi Prefecture, Japan. Patients with incomplete data or those who were transferred during treatment were excluded from the study. Patient information was collected retrospectively from electronic medical records. All 14 facilities were secondary or tertiary hospitals with inpatient facilities, ensuring no regional bias in case enrollment within the prefecture.

The diagnosis of IgA vasculitis was made according to the 2008 EULAR/PRINTO/PRES criteria if purpura or petechiae (mandatory) with lower limb predominance were present, along with at least one of the following four criteria: (1) abdominal pain; (2) histopathological confirmation of IgA deposition; (3) arthritis or arthralgia; (4) renal involvement [9].

Patients were divided into two groups according to the methods of steroid administration. The standard (STD) group was defined as patients who received steroid therapy for at least 8 consecutive days from the start of administration. The early discontinuation attempt (EDA) group was defined as patients who attempted to discontinue steroid therapy within 7 days of starting steroid therapy. Patients who had at least one steroid-free day within the first 7 days of steroid administration were categorized as the EDA group. Furthermore, patients in the EDA group were subdivided into two groups: the early discontinuation (ED) group, defined as patients who successfully completed steroid treatment within 7 days, and the readministration (RA) group, defined as patients who were unable to complete steroid treatment within 7 days and required readministration of steroids. First, we compared patient outcomes between the STD and EDA groups. Second, we evaluated total steroid dosage and length of hospitalization among the STD, ED, and RA groups. Third, we compared the outcomes of the RA and STD groups to determine whether patients in the EDA group, who required repeat steroid doses and were unable to complete the initial therapy, experienced a higher rate of adverse events and steroid-related complications. Additionally, we examined the factors influencing the steroid dosage administered to patients.

The following data were collected: patient background (age, gender, family history, history of prior infection), clinical symptoms (severity of abdominal pain, presence of grossly bloody stools), laboratory findings (blood tests, urinalysis, fecal occult blood), and outcome (PSL-equivalent steroid dosage [10], length of hospital stay, time to resolution of each symptom, complications, relapse, recurrence, and adverse effects of steroid).

The severity of abdominal pain was defined as described in a previous study [4]. Abdominal symptoms were classified in terms of severity as follows: 0 = no pain; 1 = mild pain, the child can move around and play; 2 = moderate pain, the child occasionally prefers to stay still; and 3 = severe pain, the child cannot move around or play or prefers to stay in bed. Moderate to severe abdominal pain was defined as “uncontrolled abdominal pain.” Intestinal intussusception was defined as requiring intervention, such as enemas or surgery. Proteinuria and hematuria were both defined as 1 + or higher on dipstick testing. Severe renal involvement was defined by macroscopic hematuria, massive proteinuria, nephritic syndrome, and nephritic syndrome with or without acute kidney injury. The definition of recurrence or relapse was based on a previously published study by Lei et al. [11]. Recurrence was defined as the reappearance of IgA vasculitis symptoms after a symptom-free period of at least 2 weeks but less than 3 months. Relapse was defined as the reappearance of IgA vasculitis symptoms after a symptom-free period of at least 3 months.

### Statistical analysis

Two-group comparisons were conducted to evaluate the background characteristics and results of the STD and EDA groups, as well as the steroid-related complications and adverse events between the STD and RA groups. To analyze total steroid dosage and the length of steroid administration, we used the Dunnett method following one-way ANOVA, with the STD group serving as the reference. Additionally, we conducted a univariate analysis to identify factors affecting total steroid dosage, followed by a multivariate linear regression analysis using factors that were significant in the univariate analysis. All statistical tests were two-sided, with statistical significance set at *p* < 0.05. For the univariate analysis, the chi-square test or Fisher exact test was used for categorical variables, while the Wilcoxon test was used for continuous variables. Associations among continuous variables were analyzed using Spearman’s correlation coefficient. Statistical analysis was performed using JMP version 14 and SPSS version 28.0.

### Ethics approval declaration

This study was conducted by the Declaration of Helsinki and was approved by the Nagoya University Ethics Committee (approval no. 2019–0394).

## Results

### Patient characteristics

**Fig. 1:**
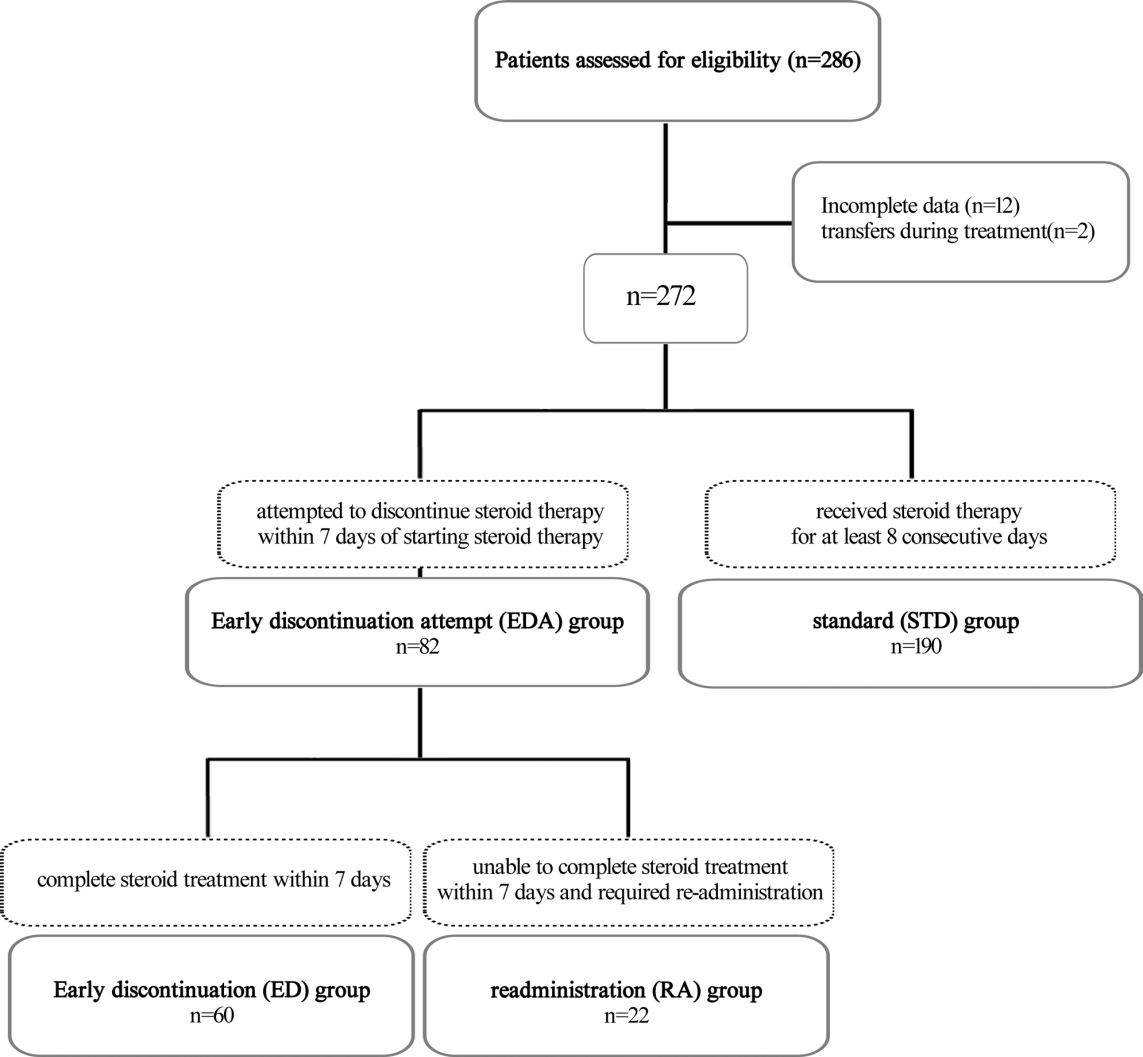
Flowchart of study patients

A total of 286 patients were initially included in the study, with 20 patients being excluded (12 with incomplete data; two transferred to other facilities during treatment). The remaining 272 patients were analyzed and divided into two groups: the EDA group (*n* = 82) and the STD group (*n* = 190) (Fig. 1).

There were no significant differences in background characteristics, including age, gender, family history, medical history, or prior infection, between the EDA and STD groups (Table 1). In all patients, steroids were administered when abdominal pain was moderate to severe. The severity of abnormal pain and the presence of grossly bloody stools before steroid initiation did not differ between the two groups. Two patients in the STD group developed intestinal intussusception before steroid initiation, whereas none did in the EDA group. No patient in either group developed intestinal perforation. Blood and urine test results, including C-reactive protein and D-dimer levels, showed no significant differences between the two groups. Acetaminophen was used as a non-steroidal drug in 40 patients (48.8%) in the EDA group and 80 patients (42.1%) in the STD group. H_2_ blockers were used in 18 patients (22.0%) in the EDA group and 16 patients (8.4%) in the STD group. The median follow-up period was 175 days (range, 2–1857) in the EDA group and 215 days (range, 0–1873) in the STD group.

**Table 1:** Patient characteristics

	**EDA (*N* = 82)**	**STD (*N* = 190)**	***p* value**
**Age at diagnosis (years), median (range)**	5.7 (2.4–13.9)	6 (2.5–14.4)	0.40
**Sex**			
Female/male, *n* (%)	43/39 (52.4/47.6)	81/109 (42.7/57.3)	0.15
**Family history**			
Renal disease, *n* (%)	1 (1.2)	5 (2.2)	0.67
IgA vasculitis, *n* (%)	3 (3.7)	2 (1.8)	0.16
**Comorbidities**			
Allergic rhinitis, *n* (%)	6 (7.3)	14 (7.4)	1.00
Atopic dermatitis, *n* (%)	4 (4.9)	6 (3.2)	0.50
Food allergies, *n* (%)	5 (6.1)	7 (3.7)	0.36
Asthma, *n* (%)	4 (4.9)	6 (3.2)	0.50
**Prior infection**			
Sinusitis, *n* (%)	3 (3.7)	11 (5.8)	0.56
Upper airway inflammation, *n* (%)	25 (30.5)	53 (27.9)	0.66
Otitis media, *n* (%)	2 (2.4)	7 (3.7)	0.73
Group A hemolytic *Streptococcus* infection, *n* (%)	18 (22.0)	26 (13.7)	0.11
**Symptoms before steroid initiation**			
Moderate abdominal pain, *n* (%)	42 (51.2)	112 (58.9)	0.29
Severe abdominal pain, *n* (%)	40 (48.8)	78 (41.1)	0.29
Grossly bloody stools, *n* (%)	9 (11.0)	32 (16.8)	0.27
Intestinal intussusception, *n* (%)	0 (0)	2 (1.1)	1.00
Intestinal perforation, *n* (%)	0 (0)	0 (0)	-
Arthritis or arthralgia, *n* (%)	59 (72.0)	123 (64.7)	0.26
**Laboratory data**			
Proteinuria*, *n* (%)	19 (23.5)	33 (17.7)	0.31
Hematuria*, *n* (%)	13 (16.1)	20 (10.7)	0.23
CRP (mg/dL), median (range)	0.7 (0–9.6)	0.6 (0–10.7)	0.09
Neutrophil–lymphocyte ratio, median (range)	3.3 (0.4–10.9)	2.9 (0.2–14.2)	0.20
IgA (mg/dL), median (range)	158 (30–476)	171 (52–479)	0.61
Factor XIII level (%), median (range)	74 (27–158)	70 (10–139)	0.15
D-dimer (µg/dL), median (range)	6.7 (0.9–31.2)	7.1 (0.1–140.3)	0.83
FDP (µg/dL), median (range)	14.9 (2.5–47.1)	14.4 (2.1–345)	0.55
**Medications other than steroids**			
Acetaminophen, *n* (%)	40 (48.8)	80 (42.1)	0.35
H_2_ blockers, *n* (%)	18 (22.0)	16 (8.4)	< 0.01
**Follow-up period, days, median (range)**	175 (2–1857)	215 (0–1873)	0.06

*EDA* early discontinuation attempt, *STD* standard, *CRP* C-reactive protein, *IgA* immunoglobulin A, *FDP* fibrin degradation products

^*^Proteinuria and hematuria were both defined as 1 + or higher on dipstick testing

### Treatment outcomes in the EDA and STD groups

We evaluated the impact of two steroid administration regimens on time to symptom resolution, length of hospital stay, steroid dosage, complications, and recurrence or relapse (Table 2). In the EDA group, the initial steroid dose ranged from 0.3 to 2.3 mg/kg/day (1–3 divided doses), with a median of 1.0 mg/kg/day. In the STD group, it ranged from 0.3 to 2.5 mg/kg/day (1–4 divided doses), with a median of 1.0 mg/kg/day. There was no significant difference in the number of days from steroid administration to the resolution of abdominal pain, grossly bloody stools, purpura, or arthritis or arthralgia between the two groups. However, the median length of hospital stay was significantly shorter in the EDA group (8.5 days vs. 15.0 days in the STD group, *p* < 0.01). Similarly, the total duration of steroid administration was significantly shorter in the EDA group (6 days vs. 17.5 days in the STD group, median, *p* < 0.01), and the total steroid dosage (PSL equivalent) was significantly lower in the EDA group (5.4 mg/kg vs. 15.4 mg/kg in the STD group, median, *p* < 0.01). Regarding severe clinical manifestations, there were two cases of intestinal intussusception in the STD group, but none in the EDA group. Both cases were successfully reduced using air or gastrografin enemas, and neither required surgery. There was no significant difference between the two groups in the incidence of intestinal perforation or severe renal involvement, recurrence, or relapse.

**Table 2:** Outcomes of the EDA and STD groups

Outcomes	**EDA (*N* = 82)**	**STD (*N* = 190)**	***p* value**
**Duration from steroid administration to the resolution of the symptom (days)**			
Abdominal pain, median (range)	7 (1–132)	9 (1–134)	0.29
Grossly bloody stools, median (range)	2 (1–20)	1 (1–27)	0.76
Purpura, median (range)	19 (1–227)	27 (1–442)	0.11
Arthritis or arthralgia, median (range)	4 (1–80)	6 (1–371)	0.19
**Length of hospital stay (days), median (range)**	8.5 (2–53)	15.0 (3–97)	< 0.01
**Steroid administration**			
Total duration (days), median (range)	6 (1–38)	17.5 (8–126)	< 0.01
Disease duration of last dose (days), median (range)	7 (1–40)	17 (8–146)	< 0.01
PSL equivalent initial steroid dose (mg/kg/day), median (range)	1.0 (0.3–2.3)	1.0 (0.3–2.5)	0.13
PSL equivalent total steroid dosage (mg/kg), median (range)	5.4 (0.4–41.3)	15.4 (4.3–89.6)	< 0.01
**Severe clinical manifestations after steroid initiation**			
Intestinal intussusception, *n* (%)	0 (0)	2 (1.1)	1.00
Intestinal perforation, *n* (%)	0 (0)	0 (0)	-
Severe renal involvement, *n* (%)	4 (4.9)	14 (7.5)	0.60
**Recurrence**, *n* (%)	7 (8.9)	12 (6.7)	0.61
**Relapse**, *n* (%)	2 (2.6)	10 (5.5)	0.52

*EDA* early discontinuation attempt, *STD* standard, *PSL* prednisolone

### Steroid-induced adverse events in the EDA and STD groups

We examined whether different methods of steroid administration affected the incidence of steroid-induced adverse events. There was no significant difference between the EDA and STD groups in the occurrence of hypertension requiring treatment, ocular hypertension, hyperglycemia, or infections that may be associated with steroid use during treatment or within 4 weeks after discontinuation (Table 3).

**Table 3:** Steroid-induced adverse events in EDA and STD groups

Adverse event	EDA (*N* = 82)	STD (*N* = 190)	*p* value
Hypertension, *n* (%)	0 (0)	3 (1.6)	0.56
Ocular hypertension, *n* (%)	1 (1.2)	4 (2.1)	1.00
Hyperglycemia,* n* (%)	0 (0)	0 (0)	-
Infections, *n* (%)	3 (3.7)	4 (2.1)	0.44

*EDA* early discontinuation attempt, *STD* standard

### Steroid readministration in the EDA and STD groups

To determine whether an early attempt to discontinue steroids resulted in unfavorable outcomes, we subdivided the EDA group into two groups: the early discontinuation (ED) group and the readministration (RA) group, as defined in the “Methods” section. In the RA group, steroids were re-administered when abdominal pain worsened to a moderate or greater intensity after steroid discontinuation. Then, we compared outcomes such as total steroid dosage, duration of administration, length of hospital stay, rates of complications, and relapse/recurrence between the RA and STD groups.

**Fig. 2:**
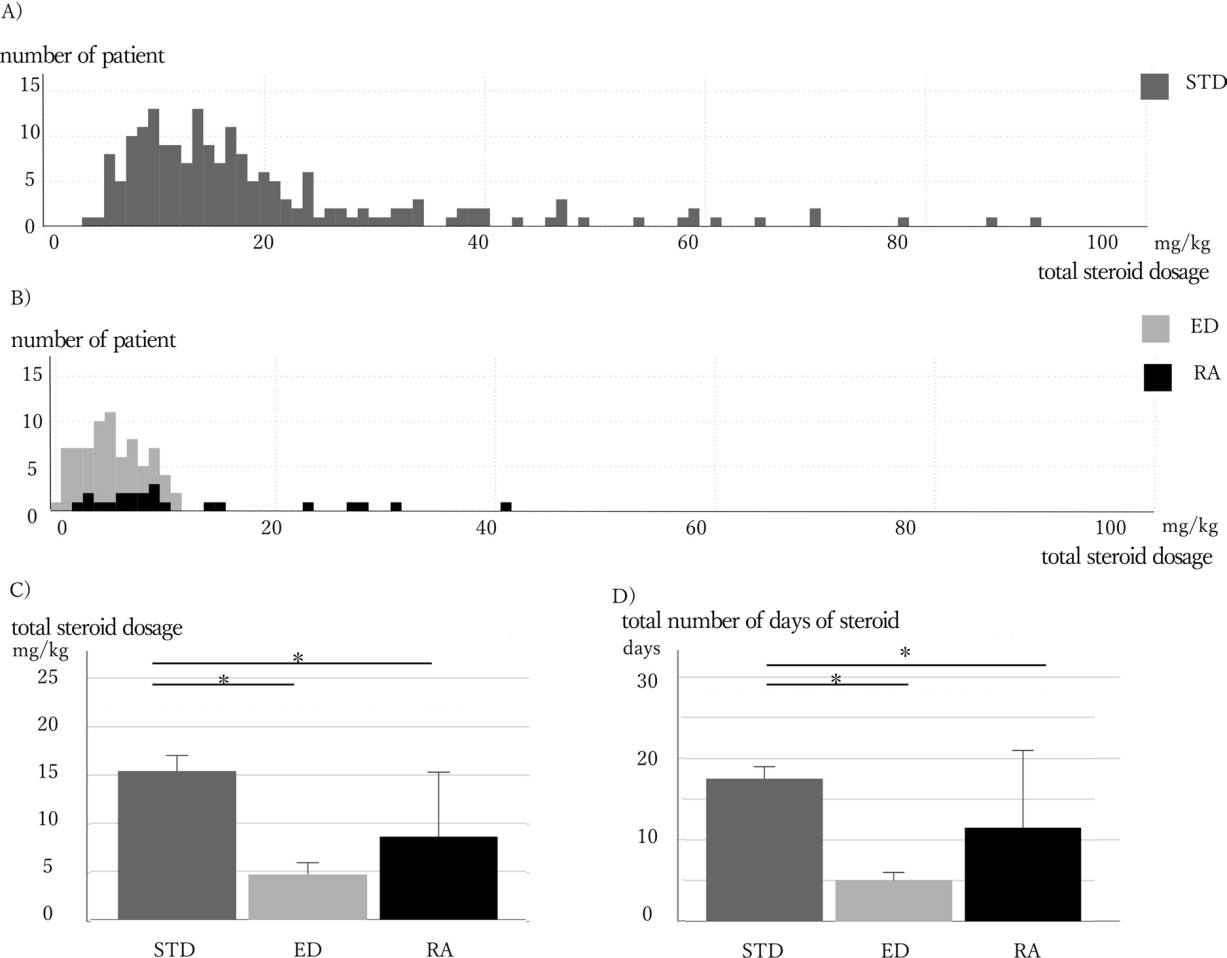
Total steroid dosage and duration in the STD, ED, and RA groups. **A** Distribution of total dosage of steroids in STD group. **B** Distribution of total dosage of steroids in ED and RA groups. **C** Median total steroid dosage comparison. The RA and ED groups have lower median steroid dosage than the STD group. Data are presented as median (95% CI; confidence interval). **D** Median of total number of days on steroids. Both the RA and ED groups received steroids for fewer days than the STD group. Data are presented as median (95% CI; confidence interval). Patients in the ED and RA groups had lower total steroid dosage compared to the STD group. EDA, early discontinuation attempt; STD, standard; ED, early discontinuation; RA, readministration; *p < 0.05

Among the 82 patients in the EDA group, 22 patients (27%) required a second course of steroids due to the reappearance of abdominal symptoms shortly after the initial discontinuation (RA group), while the remaining 60 patients (73%) did not (ED group). Figure 2A shows the distribution of patients by total steroid dosage per body weight for the STD group, and Fig. 2B provides a similar analysis for the ED and RA groups. The total steroid dosage in the ED group was significantly lower than that in the STD group (4.7 mg/kg vs. 15.4 mg/kg, median, *p* < 0.01, Fig. 2C). Notably, the total steroid dosage in the RA group was also significantly lower than that in the STD group (8.6 mg/kg vs. 15.4 mg/kg, median, *p* = 0.02, Fig. 2C). The duration of steroid administration was also significantly shorter in both the ED and RA groups compared to the STD group (5 and 11.5 days vs. 17.5 days in the STD group, median, *p* < 0.01 and *p* = 0.01, respectively, Fig. 2D).

There were no significant differences between the RA and STD groups in the length of hospital stay, incidence of severe clinical manifestations, recurrence, or relapse (Table 4). These findings indicate that even if early steroid discontinuation attempts are unsuccessful and readministration is required, patient outcomes are not adversely affected. Notably, early steroid discontinuation attempts led to reduced total steroid dosage and a shorter length of hospital stay compared to the STD group.

**Table 4:** Outcomes in the RA and STD groups

	RA (*N* = 22)	STD (*N* = 190)	*p* value
**Length of hospital stay (days), median (range)**	17 (4–40)	15 (3–97)	0.64
**Severe clinical manifestations**			
Intestinal calculus, *n* (%)	0 (0)	4 (2.1)	1.00
Intestinal perforation, *n* (%)	0 (0)	0 (0)	-
Severe renal involvement, *n* (%)	2 (9.1)	14 (7.5)	0.68
**Recurrence**, *n* (%)	1 (4.5)	12 (6.7)	1.00
**Relapse**, *n* (%)	1 (4.5)	10 (5.5)	1.00

*RA* readministration, *STD* standard

### Factors that influence steroid dosage

Finally, we analyzed factors influencing total steroid dosage. Patient background was not significantly associated with total steroid dosage (Table 5). Early discontinuation of steroids (EDA) was significantly associated with a lower total steroid dosage (5.4 mg/kg vs. 15.4 mg/kg in the STD group, median, *p* < 0.01). In contrast, the presence of grossly bloody stools and low Factor XIII were significantly associated with higher total steroid dosage. Specifically, patients with grossly bloody stools had a higher median total steroid dosage of 18.6 mg/kg (range 0.93–77.6) compared to 10.7 mg/kg (range 0.35–89.5) in patients without grossly bloody stools (*p* < 0.01). Factor XIII levels were inversely correlated with total steroid dosage, with a correlation coefficient of − 0.068 and a *p*-value of < 0.01, indicating that lower Factor XIII levels were associated with higher steroid doses.

**Table 5:** Effect of the factors associated with total steroid dosage

Nominal variables	mg/kg, median (range)		*p* value
	Yes	No	
**Sex**			
Male	12.1 (0.4–69.7)	11.6 (0.7–89.6)	0.21
**Family history**			
Renal disease	18.2 (4.8–53.9)	11.9 (0.4–89.5)	0.40
IgA vasculitis	10.6 (4.3–27.2)	12.0 (0.4–89.6)	0.79
**Comorbidities**			
Allergic rhinitis	15.7 (4.0–70.0)	11.4 (0.4–89.6)	0.23
Atopic dermatitis	8.1 (1.6–17.6)	12.1 (0.4–89.6)	0.07
Food allergies	17.5 (2.5–57.5)	11.5 (0.4–89.6)	0.18
Asthma	12.8 (0.35–24.9)	11.9 (0.7–89.6)	0.62
**Previous infection**			
Sinusitis	11.7 (2.4–60.6)	11.9 (0.4–89.6)	0.96
Upper airway inflammation	13.7 (0.7–86.4)	11.1 (3.4–89.6)	0.37
Otitis media	11.9 (6.5–86.4)	12.0 (0.4–89.6)	0.95
Group A hemolytic *Streptococcus* infection	9.5 (1.4–70.0)	12.3 (0.4–89.5)	0.08
**Symptoms**			
Severe abdominal pain	13.1 (0.94–89.5)	10.9 (0.35–70)	0.29
Grossly bloody stools	18.6 (0.93–77.6)	10.7 (0.35–89.5)	< 0.01
Proteinuria*	10.9 (0.35–77.6)	12.6 (0.66–89.5)	0.71
Hematuria*	10.6 (0.93–77.6)	12.2 (0.35–89.5)	0.53
**Early discontinuation attempt**	5.4 (0.4–41.3)	15.4 (4.3–89.6)	< 0.01
Continuous variables	*r*		*p* value
**Age at diagnosis, years**	0.039		0.52
**Laboratory data**			
CRP, mg/dL	0.008		0.15
Neutrophil–lymphocyte ratio	< 0.001		0.81
IgA, mg/dL	0.001		0.68
Factor XIII, %	− 0.068		< 0.01
D-dimer, µg/dL	0.012		0.11
FDP, µg/dL	0.009		0.20

*CRP* C-reactive protein, *IgA* immunoglobulin A, *FDP* fibrin degradation products

^*^Proteinuria and hematuria were both defined as 1 + or higher on dipstick testing

Multivariate regression analysis confirmed that grossly bloody stools and low Factor XIII levels were significantly associated with an increasing total steroid dosage, while early steroid discontinuation was significantly associated with reduced total steroid dosage (Table 6). Specifically, a 10% decrease in Factor XIII increased total steroid dosage by 1.09 mg/kg, grossly bloody stools increased it by 5.34 mg/kg, and a short-term dosage EDA decreased it by 12.2 mg/kg.

**Table 6:** Multiple linear regression analysis

	*B*	95% confidence interval		Standard error	*p* value
		Lower	Upper		
Early discontinuation attempt	− 12.2	− 16.3	− 8.12	2.07	< 0.01
Factor XIII, /10%	− 1.09	− 1.73	− 0.45	0.33	< 0.01
Grossly bloody stools	5.34	0.341	10.3	2.54	0.036

Dependent variable: total steroid dosage (PSL equivalent, mg/kg)

## Discussion

To our knowledge, this is the first report on the outcome of early discontinuation of steroids for abdominal pain in children with IgA vasculitis. We showed that 73% (60/82) of patients in the EDA group who attempted to discontinue steroid treatment within 7 days successfully completed steroid treatment without readministration, resulting in reduced total steroid dosage and a shortened hospital stay. In the RA group, which required readministration of steroids after discontinuation, there was a significant decrease in both total dosage and duration of steroid therapy, with no differences in length of hospital stay, complications, or relapse/recurrence when compared to the STD group. These findings indicate that readministration did not disadvantage patients. Therefore, attempting steroid discontinuation within 7 days may be beneficial.

Despite the widespread use of steroids for IgA vasculitis-induced abdominal pain, there are no reports examining the effect of different dosage and durations of steroid administration. In a prospective study conducted by Ronkainen et al., oral prednisone was given twice a day at 1 mg/kg/day for the first 14 days, followed by a 0.5 mg/kg/day weaning dose for 1 week, and then 0.5 mg/kg/day once a day on alternate days for an additional week [4]. In another prospective study, Huber et al. used 2 mg/kg/day oral prednisone for 7 days, followed by a weaning dose for the next 7 days [12]. In European consensus-based recommendations, prednisolone 1–2 mg/kg/day for 1–2 weeks, followed by weaning over the next few days, is recommended [13]. Expert opinions have suggested that steroids should be tapered slowly, typically over 3 to 4 weeks or more, to minimize the chance of disease rebound from rapid medication tapering [14]. Weiss et al. reported that rehospitalization tended to increase when steroids were administered for too short a duration or tapered too quickly [5]. Conversely, several studies have indicated an association between steroid use and subsequent relapse. Calvo-Río et al. conducted a retrospective study involving 417 patients with IgA vasculitis at a single institution, spanning a median duration of 12 years from 1975 to 2012. They found that corticosteroid use during the first episode of IgA vasculitis in children was associated with an increased risk of relapse [15]. Similarly, Trapani et al. reviewed cases of all children admitted to a single children’s hospital with a diagnosis of IgA vasculitis from 1998 to 2002, revealing that steroid use was associated with relapse and recommending limiting the early use of prednisone to a carefully selected group of children with IgA vasculitis [16]. Furthermore, Lei et al. conducted a large retrospective study on recurrent IgA vasculitis, examining 1002 patients under 18 years old between 1997 and 2012. They found that steroid administration for more than 10 days was a significant risk factor for relapse [11]. Given these findings, the use of steroids and the duration of their administration should be carefully considered. Nevertheless, there is still insufficient evidence to support an association between steroid administration and adverse events such as relapse and recurrence due to the retrospective nature of these studies, the small number of cases, and the lack of detailed data on steroid administration methods. Our findings suggest that short-term administration of steroids for IgA vasculitis may reduce the total steroid dose and relapse.

Contrary to our findings, it could be argued that the patients who discontinued steroids within 7 days were likely mild cases. In our study, however, there was no significant difference in patient background, such as symptoms, and laboratory data in STD and EDA groups, suggesting similar disease severity. More steroids may be needed in more severe cases, but if symptoms improve regardless of initial disease severity, short-term administration of steroids should be considered.

In analyzing factors associated with total steroid dosage, grossly bloody stools and a decrease in Factor XIII were associated with an increase in total steroid dosage. On the other hand, EDA was associated with a reduction in total steroid dosage. These results remained significant after multivariate analysis, indicating that attempting to discontinue steroid administration within 7 days, even after adjusting for Factor XIII and grossly bloody stools, reduces total steroid dosage. These results also suggest that attempting to discontinue steroid administration within 7 days in patients without grossly bloody stools and Factor XIII decline may reduce the total steroid dosage and shorten the hospital stay.

Strengths of the study include the large number of participating cases, the involvement of multiple centers, the low sampling bias due to the all-case survey, and the high follow-up rate. According to available literature, this is the first study to examine the dosage and duration of steroid administration for abdominal pain due to IgA vasculitis in children. Our findings provide a foundation for further prospective studies aimed at identifying optimal steroid administration strategies. A limitation of our retrospective study is the unequal sample size between the groups: the number of patients in the EDA group was less than half the size of the STD group, and the RA group was even smaller. This disparity may reduce the statistical power of our comparisons. Additionally, it is possible that patients with more severe conditions were more likely to be classified in the STD group, although no significant differences were observed in patient characteristics.

In conclusion, attempts to discontinue steroids within 7 days for abdominal pain in children with IgA vasculitis reduced the total steroid dosage without an increase in complications and contributed to a shorter hospital stay, even if readministration was required.

## Data Availability

No datasets were generated or analysed during the current study.
